# Clinical, radiological, and laboratory features of HIV-negative pulmonary cryptococcosis with regard to serum lateral flow assay

**DOI:** 10.3389/fmed.2024.1234474

**Published:** 2024-05-16

**Authors:** Jiejun Shi, Jianhua Chen, Liqing Hu, Qifa Song, Guoqing Qian

**Affiliations:** ^1^Department of Infectious Diseases, The First Affiliated Hospital of Ningbo University, Ningbo, Zhejiang, China; ^2^Department of Radiology, The First Affiliated Hospital of Ningbo University, Ningbo, Zhejiang, China; ^3^Department of Clinical Laboratory, The First Affiliated Hospital of Ningbo University, Ningbo, Zhejiang, China; ^4^Medical Data Research Center, The First Affiliated Hospital of Ningbo University, Ningbo, Zhejiang, China

**Keywords:** lateral flow assay, HIV-negative, pulmonary cryptococcosis, serum cryptococcal antigen, solitary nodule

## Abstract

**Introduction:**

Cryptococcosis is the second most common invasive yeast infection in China. Pulmonary cryptococcosis (PC) is difficult to diagnose due to the lack of specific clinical features and the limitation of diagnostic techniques. Although lateral flow assay was very useful in diagnosing cryptococcal infection, quite a few patients with PC presented negative serum lateral flow assay (sLFA).

**Methods:**

We conducted a retrospective study of HIV-negative patients who were diagnosed with PC in our hospital over the past decade to explore the potential relationship between the clinical profiles and sLFA in PC.

**Results:**

In total, 112 patients with sLFA tested were enrolled in this study, of which 58.93% were male. The positivity rate of sLFA for PC was 91.07%. The extent of pulmonary lesions was positively correlated with sLFA grade (Spearman *r* = 0.268, *p* < 0.01). Solitary nodule (SN) and pneumonia were the most common imaging findings in PC with negative and positive sLFA respectively. Among 65 symptomatic PC patients, 14 presented with fever and had higher hypersensitive C-reactive protein (hsCRP) level and more extensive pulmonary involvement (Mann-Whitney U test, *p* < 0.05) than those without fever. Symptomatic PC patients were more likely to have positive results of sLFA (Mann-Whitney U test, *p* = 0.05) compared against asymptomatic ones.

**Discussion:**

In conclusion, negative sLFA cannot exclude PC in patients with a solitary nodule in lung. Positive sLFA is more reliable in diagnosing PC in symptomatic patients with diffused lesions in lung who generally experience a more severe systemic inflammatory reaction.

## 1 Introduction

Pulmonary cryptococcosis mainly caused by inhalation of cryptococci spore can affect both immunocompetent and immunocompromised patients (1). The radiological manifestations of PC are relatively non-specific and can be easily mistaken for other pulmonary diseases such as cancer, tuberculosis, and bacterial pneumonia (2). Traditional blood culture is more useful for diagnosing disseminated cryptococcosis than PC (3). Furthermore, blood culture for fungi is time-consuming and has a low detection rate. Pathological examination is one of the gold standards for diagnosing PC (4). However, obtaining biopsy specimens through invasive procedures carries risks and might be rejected by some patients.

Cryptococcal antigen (CrAg) tests demonstrated excellent sensitivity and specificity in diagnosing cryptococcosis in clinical practice (5–7). Positive CrAg in cerebrospinal fluid (CSF) is one of the diagnostic criteria for cryptococcal meningoencephalitis (CM), as recommended by the guidelines (8). Furthermore, the sensitivity and specificity of serum CrAg in CM patients with HIV were found to be very high (99.7 and 94.1%) (5). However, a significant proportion of PC patients, particularly those with a localized lesion in the lung, tested negative for serum CrAg (9, 10). Previous research showed serum CrAg was false-negative in 35.45% of PC patients (11) of whom 61.19% were asymptomatic. However, some studies showed the converse findings. It was reported that serum CrAg titer could dynamically monitor the antifungal effect in PC (12, 13). Thus, the relationship between serum CrAg and PC clinical profile is still ambiguous. In this study, we aim to explore the correlation among serum CrAg, radiological features, and clinical presentation of PC. Our findings may contribute to improving the diagnostic accuracy of PC and guiding clinical decision-making.

## 2 Methods

### 2.1 Ethics statement

The research was approved by the Ethics Committee of Ningbo First Hospital (NO. 2020-R053). Informed consent was not required since this is a retrospective study. The privacy information of participants was hidden in this research.

### 2.2 Study design

#### 2.2.1 Enrollment of participants

The medical records of patients with pulmonary cryptococcosis (PC) who were admitted to our hospital from 2011 to 2020 were reviewed. We searched our electronic inpatient medical system for the discharge diagnosis of PC and enrolled patients who met the inclusion criteria but did not meet the exclusion criteria.

Inclusion criteria: Patients who met both of the following two criteria were included in this study (8).

1. At least one of the following conditions: positive sLFA; culture of BALF isolated Cryptococcus; PC was confirmed histologically.
2. Newly developed pulmonary lesions on CT scan.

Exclusion criteria: participants who did not meet the following items were excluded.

1. Postoperative pathology confirmed other lung diseases.
2. Coinfected by HIV.
3. Extrapulmonary involvement.
4. Lack of sLFA result.
5. Younger than 14 years.

Patients with at least one of the following diseases were regarded as immunocompromised: diabetes, chronic kidney disease, chronic viral hepatitis B, malignancy, autoimmune disease, tuberculosis, or long-term use of immunosuppressants.

### 2.3 Laboratory and radiological examination

The LFA test was conducted using a Cryptococcus antigen detection kit (IMMY, USA). The protocol can be referred to for detailed operation and result illustration. Results were read 10 min after adding the sample. LFA grade was defined as positive, weak positive, and negative. A weak positive indicates two bands with a lighter color. Culture for fungi was conducted on glucose agar culture medium at 37°C for 5–7 days.

The extent of pulmonary lesions was categorized as follows: solitary nodules, scattered lesions in a single lung lobe, diffuse lesions in a unilateral lung, or diffuse lesions in bilateral lungs.

### 2.4 Pathological examination

Tissue was obtained from the pulmonary lesion via transbronchial lung biopsy, thoracoscopic surgery, or CT-guided lung puncture. All the biopsy specimens were processed through chemical fixation, paraffin embedding, and special stains including hematoxylin–eosin (HE), Grocott’s methenamine silver (GMS), and periodic acid–Schiff (PAS), followed by microscopy.

### 2.5 Statistical analysis

We used SPSS Statistics 25 software (IBM, Armonk, NY, USA) to analyze the data. Numerical data were expressed as mean and standard deviation. Hierarchical data were analyzed via the Mann–Whitney U test. Correlations analysis was conducted using Spearman’s rank correlation. Categorical data were compared using the chi-squared test, and α segmentation was used for pairwise comparison between data.

## 3 Results

A total of 206 patients with a discharge diagnosis of PC were initially identified from the medical records system. However, after applying the inclusion and exclusion criteria, 112 patients were finally included in the study. Among them, 66 (58.93%) were male, and the mean age was 57.9 ± 13.2 years. The demographic and clinical characteristics of the patients are summarized in Table 1. All the participants were divided into three groups based on their sLFA level: the sLFA-negative group (*n* = 11, 9.82%), the sLFA-weak positive group (*n* = 20, 17.86%), and the sLFA-positive group (*n* = 81, 72.32%) (Table 1). No significant differences were observed in sex, age, residence, drinking, smoking, and basic diseases among the three groups. In the sLFA-negative group, PC was diagnosed by biopsy pathology (60%) and BALF culture for Cryptococcus (40%). All the participants had negative blood cultures for Cryptococcus.

**Table 1:** Correlation between serum lateral flow assay and clinical data.

	sLFA			M-W U	*p* value
	Positive	Weak positive	Weak negative		
Age(mean ± SD)	47.66 ± 13.08	45.55 ± 15.5	49.9 ± 10.42		>0.05*
Patients (Male)	82 (49)	20 (14)	10 (3)	1,440	0.552
Smoking	6	1	1	838.5	0.261
Drinking	18	0	3	413	0.965
Residence (City)	51	15	5	1425.5	0.815
Basic diseases	30	10	3	1,392	0.545
Diabetes	4	4	0	332	0.221
AID	11	4	2	726.5	0.397
CHB	8	0	0	296	0.08
Malignancy	9	4	1	619.5	0.779
Tuberculosis	0	1	0		
CKD	4	2	1	304.5	0.329
Symptoms	52	9	4	1275.5	0.055
Fever	10	4	0	680	0.946
Cough	39	6	4	1378.5	0.212
Expectoration	28	5	2	1209.5	0.264
Chest pain	6	2	0	398	0.793
Dyspnea	8	1	0	379.5	0.246
Exposure△	8	1	0		

The Mann–Whitney U test was conducted on sLFA grade between two groups divided by sex, smoking, drinking, residence, basic diseases, and symptoms, respectively.

^*^LSD test for post-hoc comparisons.

△: Definite contact with plant or pigeon droppings.

M-W U, Mann–Whitney U test; CHB, chronic viral hepatitis B; AID, autoimmune disease; CKD, chronic kidney disease; SD, standard deviation.

Among the 112 patients, 47 (41.96%) were asymptomatic and 65 (58.04%) were symptomatic. Some patients had two or more symptoms. Sixty patients presented respiratory symptoms, which were the most common symptoms observed in PC, and 14 patients presented with fever. Symptomatic PC patients were more likely to have positive results of sLFA (Mann–Whitney U test, *p* = 0.055) and higher levels of hs-CRP, white blood cell count, and serum sodium level(Mann–Whitney U test, *p* < 0.05), as compared to asymptomatic ones. Furthermore, patients with fever had higher hs-CRP levels (Mann–Whitney U test, *p* < 0.001) and more extensive pulmonary involvement (Mann–Whitney U test, *p* = 0.036) than afebrile ones.

**Figure 1:**
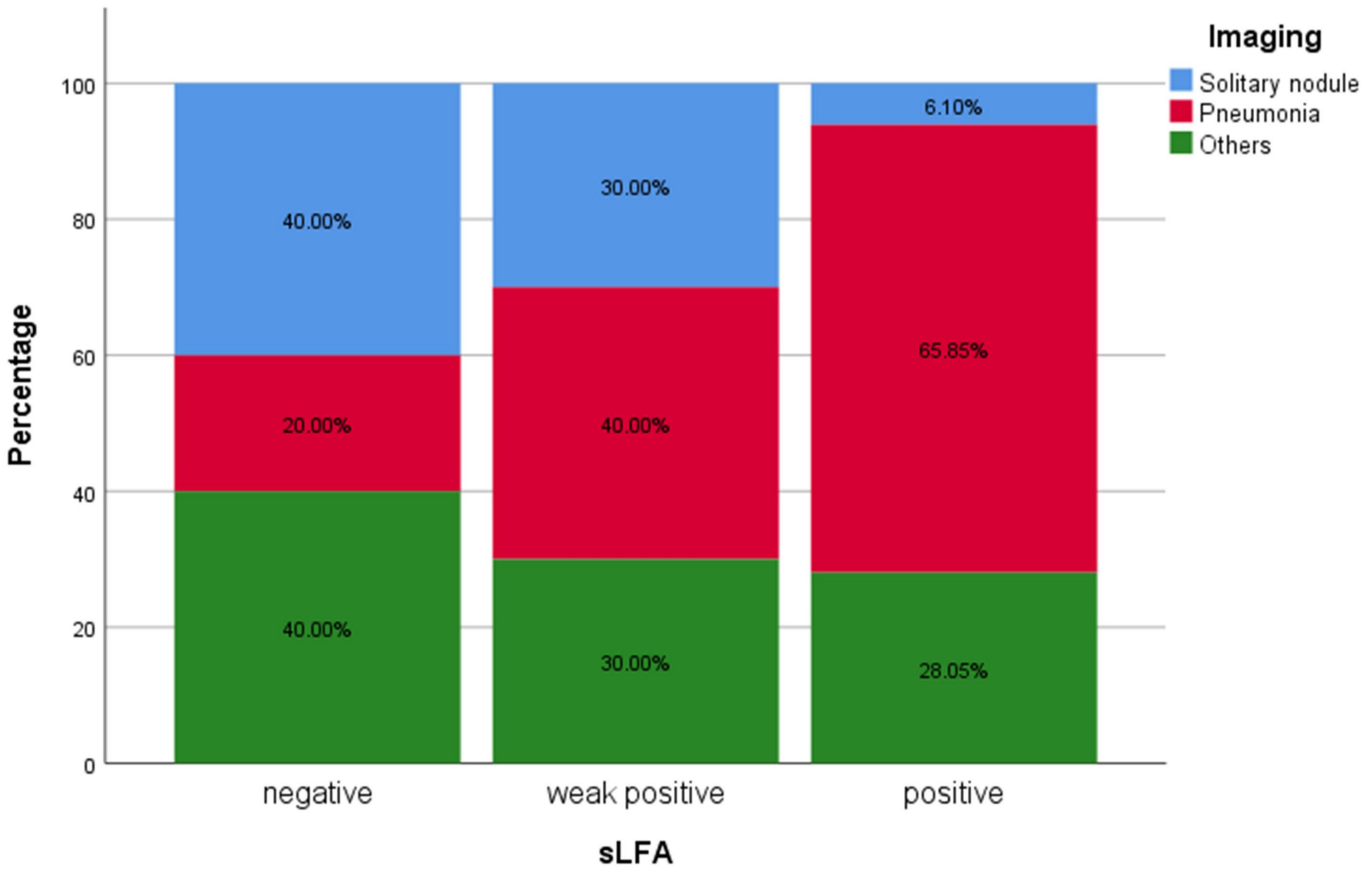
Percentages of different image features in each group classified by serum lateral flow assay rank. The proportions of patients with different image features in specific LFA groups were displayed on *Y*-axis. Overall, there were 10 patients in negative sLFA group, 20 patients in weak positive sLFA group and 82 patients in positive sLFA group.

**Figure 2:**
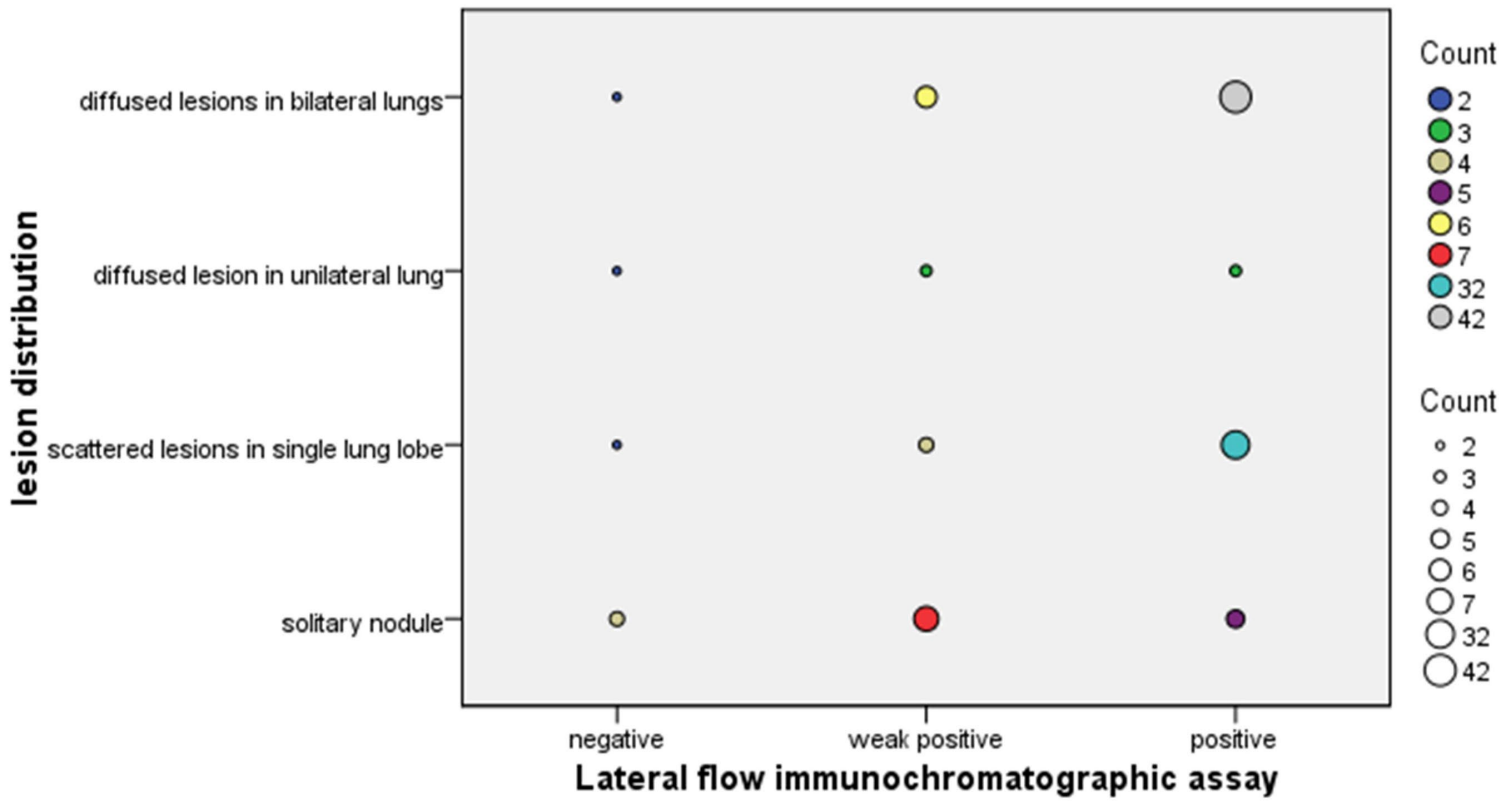
Differential lesion distributions in the lung by serum lateral flow assay grade in PC patients. The size of the dots is proportional to the number of patients. Different colours represent different number of patients which has been marked in the legend on the right side of the picture.

Regarding radiography, the most common finding in imaging in the sLFA-positive group was pneumonia (Figure 1). SN was significantly less common in in sLFA-positive patients (*p* < 0.017) compared to sLFA-negative or weak positive patients (Table 2). The extent of pulmonary lesions was positively correlated with sLFA grade (Spearman’s *r* = 0.268, *p* = 0.004) (Table 3). The distribution of the population with different extents of pulmonary lesions in the three groups is shown in Figure 2.

**Table 2:** Comparison of radiological characteristics between any two groups categorized by sLFA grade.

	Solitary nodule		*p*-value	Pneumonia		*p*-value
	0	1		0	1	
sLFA-P	77	5	0.005*	28	54	0.034
sLFA-WP	13	6		12	8	
sLFA-N	6	4	0.698	8	2	0.419
sLFA-WP	13	6		12	8	
sLFA-P	77	5	0.007*	28	54	0.014*
sLFA-N	6	4		8	2	

*p < 0.017 was regarded as a significant difference due to *α* segmentation.

sLFA-P, positive serum LFA; sLFA-N, negative serum LFA; sLFA-WP, weak positive serum LFA.

**Table 3:** Correlation between serum lateral flow assay and pulmonary lesions.

	sLFA			Spearman’s *r*	M-W U	*p* value
	Positive	Weak positive	Weak negative			
SN*	5	6	4		382.5	<0.001
PN*	54	8	2		1,116	0.001
Cavity*	16	3	1		839	0.427
Consolidation*	9	2	0		492.5	0.427
LDE^&^	–	–	–	0.268		0.004

SN, solitary nodule; PN, pneumonia; LDE, lesion distribution extent; M-W U, Mann–Whitney U test; sLFA, serum lateral flow assay.

^*^Mann–Whitney U test; & Spearman’s rank correlation.

The immune state was assessed based on preexisting diseases. In total, 43 (38.39%) patients had underlying diseases that made them immunocompromised, out of which 12 (27.91%) patients had more than one comorbidity. The most common comorbidity was an autoimmune disease, followed by malignancy, both of which frequently lead to abnormal inflammatory reactions and need long-term usage of glucocorticoids or immunosuppressive agents. No significant difference was observed in the morphology of the CT image, sLFA grade, hs-CRP level, or symptom between immunocompetent and immunocompromised hosts (all *p* > 0.05).

## 4 Discussion

The diagnosis of PC is still challenging despite the emergence of various detection technologies. Next-generation sequencing (NGS) is a promising technique that has the ability to detect all known pathogens in theory. However, a large-scale retrospective study revealed that its sensitivity is lower than that of serum CrAg tests and histopathology in immunocompetent hosts with cryptococcosis. This may be partially due to the incomplete disruption of the cryptococcal thick cell wall (14). Thus, CrAg tests such as latex agglutination (LA), LFA, glucuronoxylomannan antigen test (GXM), enzyme-linked immunosorbent assays (ELISAs) remain vital diagnostic methods for cryptococcosis. The LFA is cheaper and faster compared to LA and has a broad application prospect in low-income and resource-limited countries (15). The CSF LFA test has been proven to have a high sensitivity and specificity in patients with cryptococcal meningitis (16). However, the serum CrAg test was considered to have limited diagnostic value in PC. Henri Taelman et al. found that all PC patients without extrapulmonary involvement tested negative for serum LA (9). To check the accuracy of this view, we reviewed previous literature about pathologically confirmed non-HIV PC without extrapulmonary involvement and the performance of serum CrAg tests in PC and presented a summary in Table 4. The number of reports about pathologically confirmed pulmonary cryptococcosis infection is limited. None of the studies enrolled more than 100 participants. The sensitivity of serum CrAg tests in PC ranged from 65.9 to 97% depending on the type of CrAg test. Thus, the sensitivity of sLFA in PC may be ambiguous. In our study, we found the positivity of sLFA in PC was 91.07% which was higher than some previous reports. This may be because we excluded all the PC patients without sLFA results including 34 pathologically proven PC patients who underwent thoracoscopic surgery. Most of them presented with single nodule-mimicking tumors in chest CT and may probably have negative sLFA results, according to the regularity derived from our research. It is common for surgeons to ignore the cryptococcal CrAg test before an operation (23).

**Table 4:** Performance of CrAg tests in pathologically confirmed HIV-negative PC without extrapulmonary involvement.

Author, year	Country	Number of participants*	Specimen	CrAg tests evaluated	Sensitivity
Min et al., 2020 (17)	China	78	serum	LFA	69.23%
Zhou et al., 2018 (18)	China	89	serum	LA and LFA	93.3%
Zhu et al., 2018 (19)	China	85	serum	LFA	65.9%
Li et al., 2022 (20)	China	37	serum	GXM	97%
Liang et al., 2020 (21)	China	8	serum	Not mentioned	87.5%
Singh et al., 2008 (22)	United States, Canada, Spain, France, and India	30	serum	Not mentioned	73.3%
Zhang et al., 2012 (23)	China	7	serum	LA	71.43%

*Patients who underwent assays for CrAg. LFA, lateral flow assay; LA, latex agglutination test; GXM, cryptococcal glucuronoxylomannan antigen test.

Importantly, we discovered a positive correlation between the extent of PC lesion and the sLFA grade. High serum CrAg titer reflects high fungi load (24) which can induce diffuse pulmonary lesions (19). Fisher (25) suggested that immunocompromised patients who have diffuse PC but do not have extrapulmonary cryptococcosis may have positive serum cryptococcal antigen detected by the LA test which is consistent with our findings. We also observed that patients with SN generally presented with negative or weak positive sLFA, which is consistent with previous findings of Zhu et al. (19). The serum CrAg test has a higher sensitivity in immunocompromised hosts (13), whose cryptococcal lesions are generally more serious and disseminated (1, 26). We could not draw this conclusion due to the inadequate data of cellular and humoral immune assessment on our participants.

In our investigation, 42% of PC cases were asymptomatic, which is similar to the previous findings of Zhang et al. (23). Non-specific respiratory symptoms were common in symptomatic PC (23), but we also observed that a few PC patients presented with fever at admission. Those PC patients with fever had higher hs-CRP levels and larger pulmonary involvement (*p* < 0.05), which may indicate a risk of extrapulmonary dissemination (10). Furthermore, patients with multilobar involvement were more likely to be symptomatic as reported by Li et al. (26).

While nodules have been reported as the most common radiological feature of PC (1, 21, 27), we found that pneumonia was more common in the chest CT scans of our PC patients. This difference may be due to a large number of asymptomatic patients who found nodules during health checkups but did not undergo further examinations for Cryptococcus, opting instead for follow-up. Furthermore, quite a few pathologically confirmed PC patients were excluded due to the lack of sLFA results, despite their imaging generally presenting as a single nodule similar to malignant neoplasm. In addition, quite a few PC patients have chest CT abnormalities characterized by cavity or consolidation which may indicate a more serious pulmonary involvement (17).

Several limitations exist in our study. Since this is a retrospective research, data homogeneity is generally low. After data reduction, the sample size is not large enough to calculate a significant difference in some aspects. Large sample studies are needed to verify our findings. Since we excluded patients without sLFA data, the positivity of sLFA may be biased. Identification of Cryptococcus species was not performed since few yeasts were isolated by culture. Central nervous system involvement may be overlooked by clinicians. Future research may benefit from larger sample sizes and the application of cellular and humoral immunoassay to all enrolled patients.

In conclusion, negative sLFA results cannot rule out the possibility of Cryptococcus infection in patients with pulmonary lesions. A high serum CrAg titer may presage disseminated infection in the lungs of patients with PC.

## Data availability statement

The raw data supporting the conclusions of this article will be made available by the authors, without undue reservation.

## Ethics statement

The studies involving humans were approved by Ethics Committee of Ningbo First Hospital. The studies were conducted in accordance with the local legislation and institutional requirements. Written informed consent for participation was not required from the participants or the participants’ legal guardians/next of kin because this is a retrospective study.

## Author contributions

SJJ drafted this manuscript. JHC analyzed imaging data. LQH conducted laboratory tests. QGQ and QFS revised the manuscript. All the authors read and approved the final manuscript.

## Funding

The authors declared that financial support was received for the research, authorship, and/or publication of this article. This study was supported by the Ningbo Youth Science and Technology Innovation Leading Talent Program (2023QL055).
